# Therapeutic effect of acupuncture and moxa combustion on prostate hyperplasia

**DOI:** 10.1097/MD.0000000000030925

**Published:** 2022-10-07

**Authors:** Huajun Bo, Jisheng Peng, Minzhi Zhuang, Wenchao Qiu, Qianqian Yu, Quanbao Yao, Huazheng Liang

**Affiliations:** a Department of Traditional Chinese Medicine, Shanghai Fourth People’s Hospital, School of Medicine, Tongji University, China; b Department of Traditional Chinese Medicine, Peking University Shougang Hospital, China; c Clinical Research Center for Anesthesiology and Perioperative Medicine, Shanghai Fourth People’s Hospital, School of Medicine, Tongji University, China; d Translational Research Institute of Brain and Brain-like Intelligence, Shanghai Fourth People’s Hospital, School of Medicine, Tongji University, China; e Department of Anesthesiology and Perioperative Medicine, Shanghai Fourth People’s Hospital, School of Medicine, Tongji University, China.

**Keywords:** benign prostatic hyperplasia, combined acupuncture and moxibustion, conservative medical treatment, study protocol

## Abstract

Both acupuncture and moxibustion have been used for thousands of years in China for diverse conditions. But there are few reports on their combined effect in managing benign prostatic hyperplasia (BPH). To answer this question, we designed a prospectively study and the present protocol described details of this randomized controlled trial (RCT).

**Methods:**

In this RCT, an estimated number of 200 patients with BPH will be enrolled from Shanghai Fourth People’s Hospital, China. They will be assigned to either the combined therapy group or the conventional western medicine group in a ratio of 1:1. The International Prostate Symptom Score (IPSS) will be assessed as the primary outcome, other parameters, including the post-voiding residual urine volume, maximum flow rate (Qmax), and average flow rate (Qave), voiding time, and time to maximum flow, are secondary outcomes.

**Discussion:**

Results of this study will provide the theoretical basis for clinicians to select combined therapy or conventional western medicine treatments for BPH patients based on the efficacy of these therapies.

**Trial registration:**

chictr.org.cn, ID: ChiCTR2000030504/ChiMCTR2000003082. http://www.chictr.org.cn/edit.aspx?pid=47719&htm=4, Registered on 5th March 2020.

## 1. Introduction

Benign prostatic hyperplasia (BPH), a medical condition that is due to the excessive proliferation of the glandular epithelial and stromal cells, is common in elderly males.^[1]^ Symptoms of BPH include hesitancy, forceful urination with a slow urination stream, frequent urination, etc. Other symptoms may occur when this condition is complicated, including urgency to urinate, irritating feeling.^[2]^ The prevalence of BPH is nearly 50% among males 50 years or older^[3,4]^ and almost all males by 90.^[3]^ As the aging population surges, the incidence and the total number of BPH patients will keep rising.^[5]^

BPH is clearly associated with age and the increment of testosterone (T) and dihydrotestosterone in the body, but the etiology is still unknown. It is a consensus that excessive proliferation of the prostate stroma is the primary mechanism, which leads to the increased mass in the transition zone of the prostate, compressing the urinary tract.^[6,7]^ Others proposed that BPH is the result of prostate inflammation and altered metabolic processes due to the frequent presence of this condition with prostate inflammation and metabolic syndromes.^[8–13]^

Medical treatments for BPH are targeting the pathogenic mechanisms, such as conservative treatment with medications and surgical removal of the excessive tissue. The latter is only for refractory patients. However, none of the treatments can cure this condition and they all have side effects or complications to different degrees.

Alpha blockers are commonly prescribed to BPH patients. This type of drugs, like doxazosin and tamsulosin, can mitigate the tension of the smooth muscle cells of the prostate and the bladder,^[14]^ resulting in less constriction of the urinary tract. Consequently, symptoms are ameliorated and the urinary flow rate increased. Due to the presence of alpha receptors in the vascular system, alpha blockers have significant side effects related to blood vessel dilation, such as orthostatic hypotension, dizziness, and fatigue.

5α-reductase inhibitors are also key medicines. They can inhibit the activity of enzymes that catalyze the transition from testosterone to dihydrotestosterone. The latter can effectively suppress cell proliferation of the prostate gland and mitigate the symptoms.^[15]^ Finasteride and dutasteride are two commonly prescribed medications. Due to the importance of male hormone in sexual behavior, their side effects, such as sexual dysfunction, may stop their use for some younger patients. Reports also suggested that intake of these two medications with lower dosages is better than either of them taken alone in relieving symptoms.^[10,16]^

Phosphodiesterase type 5 inhibitors, known to increase the level of nitric oxide and to relax the urinary tract, are also used to treat BPH patients.^[17–21]^ Combination of phosphodiesterase type 5 inhibitors with alpha blockers has shown better tolerance and therapeutic effect than alpha blockers alone due to its capacity in alleviating erectile dysfunction and improving outcomes of BPH patients.^[22–25]^

Surgical therapy has its own advantage in rapidly relieving the symptoms. However, pain and other possible complications also inflict these patients.

In traditional medicine, acupuncture has been used to treat such patients in Asian countries based on the traditional Chinese medicine (TCM) theory. It is interpreted in a way that urination is relying on the “Qi” of the Kidney. In aging males, “Qi” of the kidney is supporting the urination behavior and is slowly diminished. Therefore, it takes a longer time for these patients to urinate than younger adults. As is recorded in TCM theory, “Qi” is known to circulate the blood flow. When “Qi” is weak, blood stasis will occur, which curtails the supply of nutrition and oxygen to the prostate tissue and aggravates the symptoms (see review of ref.^[26]^). Hence, it is necessary to increase “Qi” to restore the function of the prostate. A myriad of studies have been published in China testing the effectiveness of herbs, acupuncture, or moxibustion as primary therapies for BPH patients (see review of ref.^[27]^). However, the majority of these studies were case reports and few were randomized controlled trials (RCTs). Among acupuncture studies, the selection of acupoints or the use of acupuncture with herbs was the main stream research. Few studies combined acupuncture with moxibustion to treat BPH. It is, therefore, our aim to answer this question by designing an RCT to test whether the combined therapy of acupuncture and moxibustion, which is able to reduce the size of the prostate and has fewer side effects, is as effective as conventional medical treatments.

### 1.1. Our hypothesis

Combined use of moxibustion and acupuncture applied to a few acupoints that are known to increase “Qi” of the kidney will be as effective as western medical therapies in managing BPH, or even better than them.

### 1.2. Objectives

To test whether the combined therapy with acupuncture and moxibustion is as effective as conventional western medical therapies in managing BPH using an RCT.

## 2. Study design and Methods

### 2.1. Design and setting

**Figure 1.**
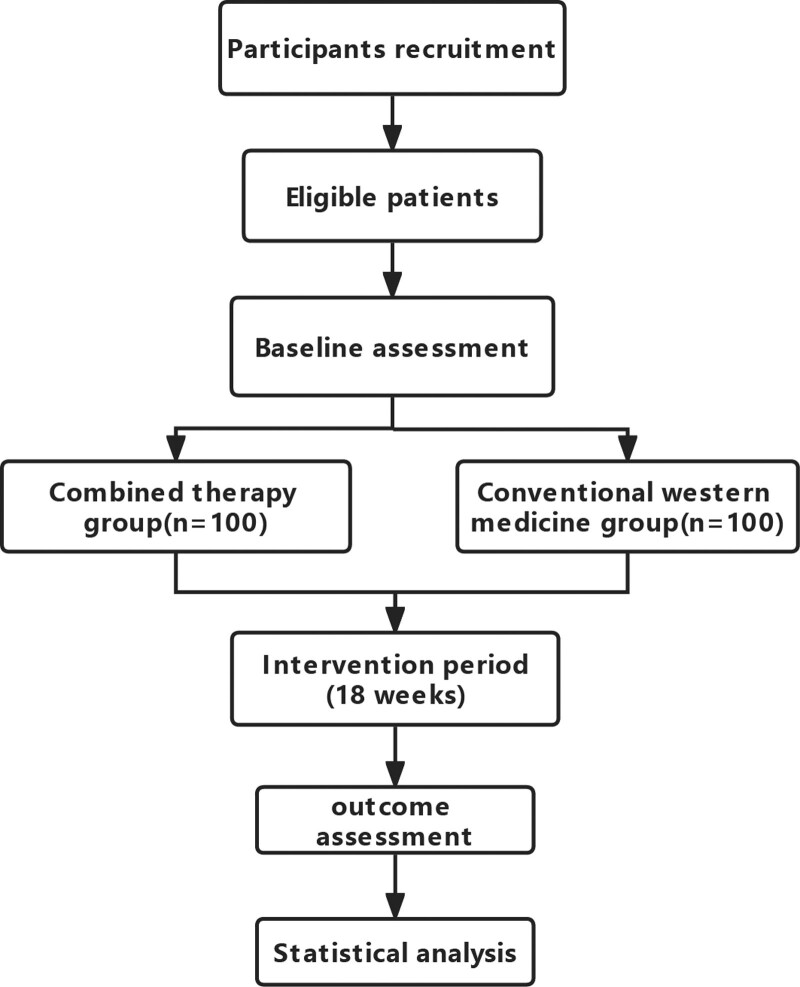
Flow chart of participants through the proposed trial.

This RCT is a single-center, investigator-initiated study, aiming to examine the efficacy of combined therapy of acupuncture and moxibustion in treating BPH. This efficacy will be compared with that of conservative western medical treatments alone. A calculated number of 200 BPH patients will be enrolled from the Urology and the Traditional Chinese Medicine clinics of Shanghai Fourth People’s hospital. BPH patients will be randomly assigned to these two groups using a random number table in a ratio of 1:1. A block size of 4 will be generated using a computer, which ensures equal numbers of patients assigned to both groups at designated time intervals. Randomization will be stratified based on the severity of symptoms, where a total score of the baseline International Prostate Symptom Score (IPSS) <7 is considered mild, 8 to 19 moderate, and 20 to 35 severe. Written informed consent to participate in this study will be obtained from all patients. The process is shown in Figure 1.

### 2.2. Participants

#### 2.2.1. Recruitment

All patients will be enrolled from the Traditional Chinese Medicine and Urology clinics of Shanghai Fourth People’s Hospital.

#### 2.2.2. Inclusion criteria

Patients will be enrolled upon meeting the following criteria:

1. Males of 45 years or older.
2. BPH diagnosed upon digital rectal examination.
3. The measured volume of the prostate ≥30 mL.
4. The maximum urinary flow rate <15 mL/second.
5. BPH patients are willing to participate in this study.
6. Continuous intake of western medicines for 3 months or more.
7. Moderate to severe BPH (IPSS score >8).

#### 2.2.3. Exclusion criteria

Patients will be excluded if they have one of the following conditions:

1. Cancer of the lower urinary tract, including prostate and bladder cancer in the past 5 years.
2. Acute urinary retention in the past 4 weeks.
3. Serious cardiovascular disorders (myocardial infarction, arrhythmia, unstable angina) in the past 6 months.
4. Concerns about the procedures, like pain due to acupuncture.
5. Calculi of the lower urinary tract, or bladder (unless completely recovered).
6. Unfit to participate in the study by investigators.
7. Use of other traditional or alternative medicines (including herbs and acupuncture) for over 1 month.
8. Sexually transmitted diseases, like syphilis, gonorrhea.
9. Congenital anomalies that affect urination.
10. Trauma or surgery that affects the lower urinary tract.
11. Upper urinary tract obstruction and other conditions that impair renal function.
12. Incapable of communicating with investigators or giving the informed consent.

### 2.3. Interventions

In the conservative medical treatment group, α-blockers, 5α-reductase inhibitors, and phosphodiesterase type 5 inhibitors are prescribed by the investigators based on individual conditions. Acupuncture or moxibustion is not allowed in this group. Patients in this group will take conventional medicines. They will be explained how the medications work and their potential side effects.

**Figure 2.**
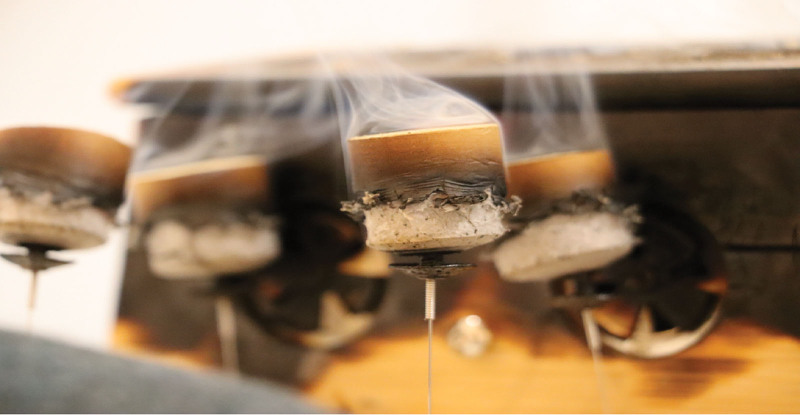
Diagram of warming acupuncture and moxibustion.

In the combined therapy group, patients will receive simultaneous acupuncture and moxibustion. The moxa segment is burnt and attached to the acupuncture needle (see Fig. 2).

Acupoints include: Zhongji (RN 3), Qihai (RN 6), Shenshu (BL 23, bilateral), Shuidao (ST 28, bilateral), Pangguangshu (BL 28, bilateral). Qihai is 1.5 *cun* inferior to the umbilicus along the anterior midline. The acupuncture needle is inserted into the body with the tip reaching a depth of 1 to 1.5 *cun*. One *cun* is defined as the width of the 1st interphalangeal joint of the patient. Zhongji is 4 *cun* inferior to the umbilicus along the anterior midline. The needle reaches a depth of 0.5 to 1*cun*. Shuidao is 3 *cun* inferior to the umbilicus and 2 *cun* lateral to the midline. The needle tip reaches 1 to 1.5 *cun*. Shenshu is 1.5 *cun* lateral to the spine of the 2nd lumbar vertebra on the back. The acupuncture needle tip reaches a depth of 1 *cun*. Pangguangshu is 1.5 *cun* lateral and inferior to the spine of the 2nd sacral vertebra on the back. The acupuncture needle tip reaches 1 *cun*. When patients have a feeling of “Teh Chi”, such as heaviness, numbness, and swelling, the needles will be kept in situ and burning moxa attached to the end of the needle. The moxa will be changed after burning out until 60 minutes are reached. These patients will be treated with the combined therapy once a week for 18 consecutive weeks. Patients in the combined therapy group will be managed by an experienced TCM doctor. Patients receiving this treatment will be managed in the same room. Communication between these patients will not impact their adherence.

Before the trial starts, patients will be briefed on our design and they have the choice to join either of the group, but which is better is not known before completing this trial. Therefore, they will be assigned to the two groups in a ratio of 1:1 after this briefing. Patients are encouraged to complete the trial with good adherence. As stated above, patients in the same group will be managed in the same room to minimize the impact of the selection of their therapies.

### 2.4. Outcome measures

**Figure 3.**
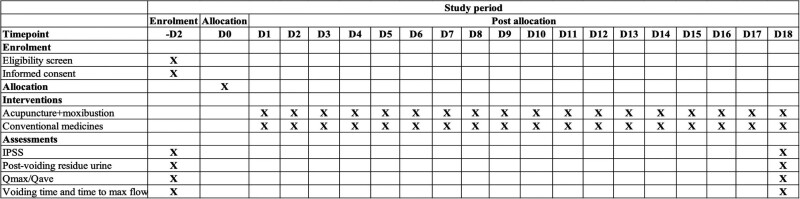
Evaluation results and time chart.

Primary and secondary outcomes will be evaluated by independent outcome assessors. They are well trained clinicians and familiar with these outcome measures and blinded to the randomization procedure. The primary outcome IPSS will be assessed using a questionnaire, secondary outcomes such as maximum flow rate (Qmax), post-voiding residual urine volume, and average flow rate (Qave), time to maximum flow, and voiding time will be measured through ultrasound examination at recruitment and 18 weeks after the trial starts. The evaluation results and time chart are as follows (Fig. 3).

#### 2.4.1. Primary outcomes

The IPSS at 18 weeks after the start of the trial will be used to assess the severity of symptoms and treated as the primary outcome. The IPSS has 8 questions with each of the first 7 questions scored 0 to 5, resulting in a sum of 0 to 35. These questions are about urinary symptoms for patients to tick one out of six answers. These include: frequency, incomplete emptying, intermittency, weak stream, urgency, straining, and nocturia. A total score of <7 is ranked mild, 8 to 19 moderate, and 20 to 35 severe. The 8th question is about the quality of life perceived by the patients, which ranges from 0 to 6, corresponding to delightful to terrible quality of life.

#### 2.4.2. Secondary outcomes

The volume of residue urine in the bladder will be measured through the ultrasound examination before and after treatment and this difference will be compared between groups.

The peak and the average urine flow rates will be calculated using the Qmax and the Qave collected from the urodynamic examination.

Voiding time, time to completely empty the bladder, and the time to maximum flow will be measured during the urodynamic examination.

### 2.5. Safety assessment

Adverse events will be closely surveilled during the treatment. If pain, bleeding, fainting, dizziness, and other symptoms occur, they will be recorded in the case report forms. They will be recorded even if these symptoms occur in patients’ houses. Responsible investigators will be immediately informed and appropriate measures taken after appropriate assessment of these patients. If the adverse events significantly impact the quality of life of these patients, they can withdraw from the study and medical care will be provided to them.

### 2.6. Sample size

To calculate the sample size, results of our pilot study were used to estimate the number of patients we need for this trial. In our RCT, the efficacy of the combined therapy and the conservative medical treatment was 80% and 60%, respectively, 0.05 for α, 0.8 for 1-β, 20% for the dropout during the follow-up. Eighty patients in each group are required as calculated using the Two Independent Proportions (Null Case) Power Analysis (Pass 11 software). A 25% dropout rate is estimated. Finally, 100 patients in each group are needed.

### 2.7. Randomization

Randomization will be conducted using a random number table to assign patients to the two groups. Firstly, block size of 4 will be generated by a computer and 200 patients will be randomly assigned to the two groups in a ratio of 1:1 after acquiring their written informed consent to join a group, which ensures equal numbers of patients assigned to both groups at designated time intervals. Patients of the mild, moderate, and severe categories will be assigned equally to the two groups.

### 2.8. Blinding

In this trial, investigators who manage patients with acupuncture and moxibustion are unaware of the allocation of patients because they will be treated with other patients in the clinic. Investigators who assess these patients during the follow-up are also blinded. The statistician is also unaware of the allocation. But patients are aware of their allocation. To minimize the impact of patients’ awareness of their allocation, they will be managed at different time points so that they are unaware of the treatment options of the other group. For patients in the same group, they will be treated in the same room and no patients from the other group will be present.

### 2.9. Statistical method

Results collected will be initially validated by two investigators before statistical analyses. Data analysis will be conducted using IBM SPSS (version 20.0) for Windows (SPSS Inc., Chicago, IL) by a statistician who is blinded to patients’ information. Continuous variables will be presented as mean ± SD or median with an interquartile range (IQR). Information of patients at enrollment will be compared using the Mann-Whitney *U* test or using the *t* test for continuous variables, and the Fisher’s exact test or χ^2^ for categorical variables. Two-side Student’s *t* test will be used to compare continuous variables like IPSS, maximum flow rate, voiding time, average flow rate, post-voiding residue urine volume, and time to maximum flow 18 weeks after treatment. Longitudinal analysis of IPSS, Qmax, voiding time, post-voiding residual urine, Qave, and time to maximum flow were done with a GLMM approach, accommodating data with nonequidistant repeated measures of time and missing values. *P* < .05 (two-sided) is considered statistically significant.

## 3. Discussion

The present trial aims to demonstrate the efficacy of the combined therapy of acupuncture and moxibustion for BPH patients, and the results will be compared with that of the conventional medical treatment.

Acupuncture has been empirically used for many medical conditions as a traditional therapeutic method. Nowadays, it is used for a myriad of chronic illnesses where western medicine does not have better options. However, the effectiveness of acupuncture is dependent on a number of factors. For example, the maneuver in punching the needles and the way to twist the needle, as well as the patients’ feeling of “Teh Chi” will determine whether the procedure is properly performed. In recent years, electroacupuncture has become a very popular device and is used for a variety of conditions due to its ease to use and consistent settings. However, manual manipulation is still the way to show the best results. Firstly, the location of acupoints vary considerably between individuals due to the difference in height and weight. Secondly, “Teh Qi” is a sensation which is essential for the confirmation of punching the right spot. Thirdly, different maneuvers lead to discrete outcomes.

According to the TCM theory, BPH is due to weakened “Qi” of the kidney. Therefore, all treatments are prescribed to replenish “Qi”, including acupuncture. On the back, two acupoints - “shenshu” and “pangguangshu”, are the most important ones for increasing “Qi” of the kidney. The abdomen has two acupoints - “qihai” and “zhongji”, both are well known for increasing “Qi” of the kidney when punched with needles. “Shuidao” is on the side of “qihai”, belonging to the Stomach meridian. In TCM practice, this acupoint is helpful for expelling the excessive water - urine. Therefore, acupuncture at these points will alleviate symptoms of BPH through different mechanisms.

Moxibustion is also a traditional therapy, especially effective for Cold conditions by increasing “Qi” and “Yang”. When the skin of acupoints is warmed by the burning moxa, it transmits the energy along the meridian it belongs to. Therefore, the combined use of acupuncture and moxibustion will synergistically increase “Qi” of the kidney and facilitate the excretion of urine, relieving the symptoms. From our experience,^[28–32]^ the combined use of acupuncture and moxibustion will have a similar efficacy to conservative medical treatments or an even better one than conservative medical treatments.

A number of clinical trials have reported that herbal decoctions are as effective as conservative medical treatments for BPH.^[33–35]^ However, individual patients have different concurrent medical issues, it is unlikely for all of them to take the same herbs. Therefore, it is very unlikely to conduct an RCT with the same herbal decoction unless a large number of patients are recruited. In contrast, it is relatively easy to prescribe the same acupoints for the complaint of patients. This renders our study a completely RCT.

Our study has limitations in a few aspects. Firstly, this is a single-center study and all patients are enrolled from the same hospital. Patient selection bias might be possible. To display the advantage of this combined therapy, moderate to severe BPH patients who might not benefit much from conservative medical treatments are recruited. Secondly, the present study has a relatively small size of patients though 100 patients are assigned to each group. It is likely that we fail to show the positive effect of the combined therapy on BPH. Thirdly, the vast majority of patients are taking conservative medicines before their enrollment. Their remaining therapeutic effect may last for a certain period after starting the combined therapy. Considering our trial will complete by the end of 18 weeks, the impact of medicines will be minimal by that time.

In summary, the proposed trial is designed to show evidence that combined use of acupuncture and moxibustion is an effective alternative therapy for BPH patients with few side effects.

## Acknowledgements

We would like to thank Prof Lize Xiong for his advice and support.

## Author contributions

HL, HB, and QY2 conceived and designed the study, QY1 contributed to the recruitment and randomization of patients, HB will treat patients with combined acupuncture and moxibustion, MZ and JP will assess patients during the follow-up, WQ will conduct statistical analysis, HB and HL drafted this manuscript.

Conceptualization: Huazheng Liang.

Data curation: Wenchao Qiu.

Formal analysis: Wenchao Qiu.

Funding acquisition: Huajun Bo.

Investigation: Huajun Bo, Jisheng Peng, Minzhi Zhuang, Qianqian Yu, Quanbao Yao.

Project administration: Huajun Bo, Minzhi Zhuang, Quanbao Yao, Huazheng Liang.

Software: Wenchao Qiu.

Writing – original draft: Huajun Bo, Jisheng Peng, Minzhi Zhuang, Qianqian Yu, Quanbao Yao, Huazheng Liang.

Writing – review & editing: Huajun Bo, Huazheng Liang.
